# Overuse, Overdose, Overdiagnosis… Overreaction?

**DOI:** 10.2349/biij.6.3.e8

**Published:** 2010-07-01

**Authors:** ELM Ho

**Affiliations:** Imaging Department, Sime Darby Specialist Centre Megah Sdn. Bhd., Petaling Jaya, Malaysia

**Keywords:** Radiation risks, medical radiation, computed tomography, radiation overuse, radiation overdose

## Abstract

When x-rays were first discovered, the harmful effects of radiation had to be manifest in the early users before they were known. Today, radiation protection and safety have been established and the effects of radiation, as well as its risks, are known. Even so, medical radiation, in particular the growth in the use of computed tomography (CT), has resulted in soaring radiation doses received by the population in general. Inappropriate use has resulted in overuse, overdose and, perhaps, overdiagnosis, especially when used in screening. In the quest to control and curb the use of procedures involving radiation, however, we must be careful not to provoke a pandemic of irrational fear of radiation. Overreaction to the overuse and overdose of radiation might deter patients from life-saving procedures.

There has been an escalation of radiation dose for medical purposes especially with the increasing use of Computed Tomography (CT) scanners [1], and hybrid modalities such as Positron Emission Tomography-CT. Heart scans for coronary artery calcifications began with the advent of the electron beam CT scanner. Today, very fast multislice or dual-source CT scanners have been added to the armamentarium to scan the heart for calcifications as well as for CT angiograms. As new indications gain acceptance, there seems to be nothing that the multislice CT scanner cannot do, particularly with further developments and the use of nano-technology such as the gold nanoprobes [2] for CT molecular imaging. The trend in Malaysia is similar, with the frequency of CT scans and interventional cardiac procedures showing a marked increase over the years [3].

With inappropriate use and overuse, comes overdiagnosed conditions which may never have become clinically significant if not discovered during screening procedures. Population-based screening mammograms have come under great scrutiny where overdiagnosis might have caused more harm than benefit [4, 5].

It is ironic, but because radiation is invisible, its potential danger is often forgotten. The discovery of the mysterious, invisible x-rays in 1895 by Wilhelm Conrad Roentgen; radioactivity in 1896 by Henri Becquerel; and radium by Marie Curie brought on a very exciting period. New-found uses of x-rays were hailed and marketed. No one knew it could cause harm, until the dangers became apparent.

That was then. Now, radiation has been well-investigated as a cause for sickness. Radiation protection was born, and radiation safety measures made this discipline harmless for its practitioners… or so we thought! Perhaps equipment has become so safe to the operators/radiologic technologists, that the potential radiation risks to patients are forgotten.

While physicians who are not radiologists or radiation oncologists can plead ignorance to the fact that radiation has potential risks, what can the latter say in their defence? In our training as radiologists, the principle of ALARA was the mantra – As Low As Reasonably Achievable. We were reminded to ensure that investigations or procedures were justified, and if so, determine the best tool to use, and to consider foremost, a tool that did not require ionising radiation (such as the ultrasound). We had to ensure that the procedure was optimised to answer the clinical question while minimising the radiation dose and still obtaining diagnostic information. After all, we studied the effects of radiation, did we not?

A breakdown in communication between referring physicians and radiologists or nuclear medicine physicians would have contributed to the increase in inappropriate, unjustified procedures in an environment of increasing workload and time pressure. Fear of litigation tends to reduce reliance on pure clinical acumen. Another contributing factor is that, increasingly, practitioners using ionising radiation are no longer just radiologists. Examples would be cardiologists and neurologists using (or self-referring) CT and Magnetic Resonance (MR) scans, as well as performing fluoroscopic-guided interventions. Then, there is also pressure from the patient as the Internet has made information – or misinformation – readily available to them.

The digital era came with exciting changes in the way we work: efficiency was increased, workflow improved, and throughput increased; yet, the lack of need for printed films (cost issues and reject films serve as constant reminders) and digital manipulation of images may have spurred radiologists and radiologic technicians (or radiographers) to slack off on diligent monitoring of radiation doses while performing procedures [6]. Dose creep is insidious but it is a real problem. Even if doses are monitored and reported by the equipment software, they often go unnoticed. This is one reason that lethal radiation therapy can be delivered “accidentally” [7].

In the last few years, journal publications and media reports have highlighted the inappropriate use of our diagnostic tools [8], and errors leading to lethal overdoses in radiation therapy. Hopefully, these reports will act as an impetus to improve healthcare delivery in these areas. The Food and Drug Administration of the USA [9] and the US Congress [10] have come into the picture, with hearings on medical radiation exposure in the first quarter of 2010. Recommendations to record radiation dose for patients over their lifetime would provide an estimate of the cumulative dose. This would guide radiologists and physicians to weigh the radiation risks with respect to cancer induction. It is now public knowledge that acute excessive radiation causing skin burns, erythema or hair loss may be seen in interventional procedures under fluoroscopic guidance or even perfusion CT of the brain. Are we overreacting to this “crisis”? Is this crisis real or perceived?

Warnings of the rising radiation dose and overuse of radiation are not new. The National Council on Radiation Protection and Measurements in 2007 reported that clinicians, including radiologists, were not cognisant of radiation exposure risks [11] and that hybrid modalities [12] such as the PET-CT would result in even higher patient radiation doses. The International Commission on Radiological Protection [13] has published reports, such as diagnostic reference levels, recommended dose limits and the biological effects of ionising radiation (BEIR). The Alliance of Radiation Safety in Paediatric Imaging developed the Image Gently Campaign [14] in 2007, and since then, there has been growing support worldwide. I am happy to state that the College of Radiology, Academy of Medicine of Malaysia is a member of this alliance; however, more measures are definitely needed to ensure that every member “lives, eats and thinks” radiation protection.

With reference to CT scanners, vendors, inventors and radiologists have collaborated to help develop protocols and to produce equipment that delivers less radiation or has software incorporated to set off an alarm to warn the users. However, machines or software cannot replace responsible, conscientious and justified use by the equipment operators, radiologists and referring physicians.

Other methods are being explored and tested, such as implementing software for decision–making [15], using appropriateness criteria developed by the American College of Radiology [16], using legislation to curb self-referrals or obtaining informed consent for every procedure [17]. In 2009, a radiology resident developed a software programme [18] for the iPhone which helps calculate doses; although the dose calculation may not directly translate into risks in such a simple manner, it serves as a guide that may come in useful for the referring physician and the patient. It also keeps track of radiation doses from procedures.

Time will tell if all the media hype and attention garnered by medical radiation will backfire in some way, unless measures are implemented carefully. The publicity and education must be communicated in ways that the layperson can understand. Otherwise, we might provoke a pandemic of irrational fear of radiation. There can be no doubt that imaging and image-guided interventions have saved many lives, perhaps more lives than they may harm. It is difficult to quantify radiation risk and to extrapolate it directly to harm when it comes to those involving mutations and cancer induction. Radiation risk is influenced by many factors, such as sex, age, organ involved and underlying genetic factors. In addition, it is not just cancer that may be induced; there are other adverse health effects to be considered as well.

While we are “battering” ourselves over the excessive doses of radiation applied in diagnostics or therapeutic, there are others out there, waiting to pounce on an opportunity to market their “alternative” imaging solutions. The screening boom for wellness has been a double-edged sword. Non-proven methods for cancer prevention have emerged, touting amazingly safe procedures that can detect signs of cancer way before cancer appears, while assuring no radiation, no pain and no side effects, or promoting the use of supplements to prevent cancers from developing. Will we be creating another problem by over-publicising the radiation risks of medical procedures and imaging tests?

Therefore, a cost-benefit-risk analysis and a balanced perspective is needed in all measures that are being taken to control medical radiation. Although there are many potential solutions, everyone must take responsibility to ensure careful implementation that is tailored for various institutions, cultures and countries. The best approach is to ask ourselves: just because a tool is there, must we use it? One size does not fit all, and no matter what, the patient’s interest comes first.

## References

[1] Brenner DJ, Hall EJ (2007). Computed tomography--an increasing source of radiation exposure. N Engl J Med..

[2] Popovtzer R, Agrawal A, Kotov NA (2008). Targeted gold nanoparticles enable molecular CT imaging of cancer. Nano Lett..

[3] Ng KH, Abdullah BJ, Sivalingam S (1999). Medical radiation exposures for diagnostic radiology in Malaysia. Health Phys..

[4] Jørgensen KJ, Gøtzsche PC (2009). Overdiagnosis in publicly organised mammography screening programmes: systematic review of incidence trends. BMJ.

[5] Kmietowicz Z (2010). Breast screening benefits twice as many women as it harms, shows new analysis. BMJ.

[6] Ng KH, Rehani MM (2006). X ray imaging goes digital. BMJ..

[7] Bogdanich W (2010). The Radiation Boom: Radiation Offers New Cures, and Ways To Do Harm. http://www.nytimes.com/2010/01/24/health/24radiation.html.

[8] Lehnert BE, Bree RL (2010). Analysis of appropriateness of outpatient CT and MRI referred from primary care clinics at an academic medical center: how critical is the need for improved decision support?. J Am Coll Radiol..

[9] (2010). FDA hearings rise above medical radiation rhetoric.

[10] (2010). Congress surprised at lack of medical radiation oversight.

[11] (2007). Report from NCRP: U.S. physicians remain oblivious to radiation exposure risks.

[12] (2007). Report from NCRP: Hybrid imaging poses radiation exposure challenges.

[13] (2003). The International Commission on Radiological Protection.

[14] Sistrom CL, Dang PA, Weilburg JB (2009). Effect of computerized order entry with integrated decision support on the growth of outpatient procedure volumes: seven-year time series analysis. Radiology.

[15] (2008). Image Gently Campaign.

[16] (2004). American College of Radiology.

[17] Picano E (2004). Sustainability of medical imaging. BMJ..

[18] (2009). iPhone/iPod Touch Program “Radiation Passport” Estimates Radiation/Cancer Risks from Radiology and Related Examinations and Procedures.

